# *Alseodaphnopsis*: A new genus of Lauraceae based on molecular and morphological evidence

**DOI:** 10.1371/journal.pone.0186545

**Published:** 2017-10-18

**Authors:** Yue-qing Mo, Lang Li, Jian-wu Li, Jens G. Rohwer, Hsi-wen Li, Jie Li

**Affiliations:** 1 Plant Phylogenetics & Conservation Group, Center for Integrative Conservation, Xishuangbanna Tropical Botanical Garden, Chinese Academy of Sciences, Kunming, P. R. China; 2 University of Chinese Academy of Sciences, Beijing, P. R. China; 3 Herbarium (HITBC), Xishuangbanna Tropical Botanical Garden, Chinese Academy of Sciences, Menglun, Yunnan, P. R. China; 4 Biozentrum Klein Flottbek, Universität Hamburg, Hamburg, Germany; 5 Herbarium (KUN), Kunming Institute of Botany, Chinese Academy of Sciences, Kunming, Yunnan, P. R. China; The National Orchid Conservation Center of China; The Orchid Conservation & Research Center of Shenzhen, CHINA

## Abstract

An investigation of a questionable species of the genus *Alseodaphne* led to the discovery of a new genus *Alseodaphnopsis* H. W. Li & J. Li, gen. nov., separated from *Alseodaphne* Nees, and a new species *Alseodaphnopsis ximengensis* H. W. Li & J. Li, sp. nov., endemic to Yunnan province, China. This new species is characterized by having big, axillary, paniculate inflorescences, as well as large, subglobose fruits. Based on DNA sequence data from two gene regions (nuclear ribosomal ITS and *LEAFY* intron II), we investigate its phylogenetic position within the *Persea* group. Phylogenies using maximum parsimony (MP) and Bayesian inference (BI) support the recognition of *Alseodaphnopsis* as a distinct genus but do not resolve well its relationship within the *Persea* group. The new genus is circumscribed, eight new combinations for its species are made, and a description and illustration of the new species are provided.

## Introduction

The *Persea* group is a subset of the Lauraceae, including seven currently recognized genera, *Alseodaphne* Nees, *Apollonias* Nees, *Dehaasia* Bl., *Machilus* Rumph. ex Nees, *Nothaphoebe* Bl., *Persea* Mill., and *Phoebe* Nees, containing a total of 400 to 450 species [1–2]. Most of these species are native to tropical and subtropical Asia, whereas ~20% are distributed in warm-temperate to tropical regions of America [1–2]. According to the results of several recent molecular studies, the *Persea* group is monophyletic [1–5]. Some of them also showed that *Persea* subg. *Eriodaphne*, *Machilus*, *Persea* subg. *Persea*, and *Phoebe* formed well-supported monophyletic groups, and the genus *Alseodaphne* was not monophyletic [1–2]. However, the relationships of species within and among the *Alseodaphne* clades are still not well resolved.

In its current circumscription, *Alseodaphne* consists of fifty species or more, of which about 90% are distributed in tropical Asia, including Cambodia, China, India, Indonesia, Laos, Malaysia, Myanmar, the Philippines, Sri Lanka, Thailand, and Vietnam; ten species (seven endemic) are present in China, including *A*. *andersonii* (King ex Hook. f.) Kosterm., *A*. *gracilis* Kosterm., *A*. *hainanensis* Merr., *A*. *hokouensis* H. W. Li, *A*. *huanglianshanensis* H. W. Li & Y. M. Shui, *A*. *marlipoensis* (H. W. Li) H. W. Li, *A*. *petiolaris* (Meisn.) Hook. f., *A*. *rugosa* Merr. & Chun, *A*. *sichourensis* H. W. Li, and *A*. *yunnanensis* Kosterm., distributed in Guangdong, Yunnan, and Hainan [6–7]. Nine of these ten species (except *A*. *hainanensis*) are distributed in the northern marginal zone of the tropics in southwestern China.

The delimitation of *Alseodaphne* Nees historically has been difficult and circumscriptions have been variable since the genus was first described by Nees [8], who incorporated four species, of which *A*. *semecarpifolia* Nees only stands. The three other species belong to *Phoebe* and *Litsea* Lam. (Lauraceae), and *Castanopsis* (D. Don) Spach (Fagaceae). Meisn. [9] followed Nees and moved *Phoebe excelsa* Nees to *Alseodaphne*. Bentham & Hooker [10] treated *Alseodaphne* and *Nothaphoebe* as sections within *Persea*. Gamble [11] recognized *Alseodaphne* and *Persea* as two distinct genera, while Hooker [12] reduced *Nothaphoebe* to *Alseodaphne*, and Boerlage [13] moved all Malesian *Nothaphoebe* to *Alseodaphne*. Ridley [14], on the other hand, kept the two genera *Nothaphoebe* and *Alseodaphne* separate in his The Flora of the Malay Peninsula. Kostermans [15] included *Alseodaphne* and *Nothaphoebe* in *Persea* in his overview of all Lauraceae, but changed his opinion and recognized *Alseodaphne* and *Nothaphoebe* as independent genera later [16]. He also thought that these two genera possibly might be fused again in the future, because they are extremely close to each other. van der Werff [17] came to a similar conclusion. He found no significant difference between the two genera and included *Nothaphoebe* in *Alseodaphne*, estimating the number of species to about 90. He also expressed doubts about the delimitation between *Alseodaphne* and *Dehaasia*. Julia et al. [18], however, recognized *Alseodaphne*, *Dehaasia* and *Nothaphoebe* as distinct genera based on a combination of numerous morphological characters. In the light of the DNA phylogenetic results, Rohwer and Rudolph [5] found that *Alseodaphne perakensis* (Gamble) Kosterm. and *Dehaasia cuneata* (Bl.) Bl. formed a strongly supported clade. Rohwer et al. [1] found that the relationship of *Nothaphoebe umbelliflora* (Bl.) Bl. and some species of *Alseodaphne* was very close, but *Alseodaphne* did not appear monophyletic in their study, although with insufficient support. The work of Li et al. [2] showed that *Alseodaphne* was clearly a polyphyletic group. Its species were placed in two distinct clades, one of which included also *Dehaasia* (5 spp. examined) and *Nothaphoebe umbelliflora*. The species of this clade are mainly distributed in tropical Asia. The second clade (Clade III in Li et al., 2011) was poorly resolved at the base and included also the species of *Phoebe* as well as a few Neotropical species currently placed in *Persea*. The *Alseodaphne* species belonging to this clade are mainly distributed in the northern margin of tropics in southwestern China. In our study, we won’t discuss in depth the relationships of species within *Alseodaphne*, *Nothaphoebe*, and *Dehaasia*, as our samples from tropical SE Asia are limited, but aim at the species of *Alseodaphne* with their main distribution in southwestern China.

We conducted an exploratory trip to Yunnan, China, and collected a questionable plant possibly belonging to *Alseodaphne*, and this was the reason to study the species from SW China in more detail. In this study, the phylogenetic relationships of the genus *Alseodaphne* distributed in southwestern China are assessed by using DNA sequence data in addition to morphological data.

## Materials and methods

### Ethics statement

Collection of these species was conducted in compliance with existing regulations for plants defined as non-commercial, as determined by local government offices. In addition, these sample collections were performed in China with the written approval from the National Forest Bureau and relevant local governments, complying with Chinese and international regulations for the collection of native plant samples.

### Morphological observations

Material of the questionable taxon was collected in November 2007, January 2008, August 2015 and November 2015 from the county of Ximeng (22°41′28.36″N, 99°38′59.68″E), Yunnan, China. The morphological description is based on fresh and pressed specimens. Details of the flowers were examined and photographed under a stereomicroscope (ZEISS Discovery V12.0). The morphological comparison with other closely related species is based on study of living plants in the field as well as herbarium specimens, supplemented by information gathered in the relevant literature [6–7,16]. The specimens examined have been deposited in the herbarium of HITBC. Inflorescence size data and fruit size data of the new taxon and known *Alseodaphne* species were collected from pressed specimens or literature searches, and compared using the independent samples *t* test. Significance was assumed at p<0.05. All statistical analyses were performed using the SPSS statistical software package 16.0 [19].

### Taxon sampling

In the present study, the ingroup sampling included 66 samples (including the new taxon) representing most of the genera within the *Persea* group (*Machilus*, *Phoebe*, *Dehaasia*, *Nothaphoebe*, and *Alseodaphne*). As in the work of Li et al. [2], nine species from four closely related genera (*Actiondaphne*, *Lindera*, *Litsea*, *Neolitsea*) were selected as the outgroups. Voucher information and GenBank accession numbers are listed in S1 Table.

### Nomenclature

The electronic version of this article in Portable Document Format (PDF) in a work with an ISSN or ISBN will represent a published work according to the International Code of Nomenclature for algae, fungi, and plants, and hence the new names contained in the electronic publication of a PLOS ONE article are effectively published under that Code from the electronic edition alone, so there is no longer any need to provide printed copies.

In addition, new names contained in this work have been submitted to IPNI, from where they will be made available to the Global Names Index. The IPNI LSIDs can be resolved and the associated information viewed through any standard web browser by appending the LSID contained in this publication to the prefix http://ipni.org/. The online version of this work is archived and available from the following digital repositories: PubMed Central, LOCKSS.

### DNA extraction, PCR amplification and sequencing

Total genomic DNA was extracted from silica-gel dried leaf specimens using the Plant Genomic DNAKit (Tiangen Biotech, Beijing, China). The analyses presented here used the sequence data from two DNA regions, the internal transcribed spacer (ITS) region (ITS1-5.8S-ITS2) of the nuclear ribosomal DNA and *LEAFY* intron II. These regions have been shown to be valuable in phylogenetic studies within the *Persea* group [2]. The ITS and *LEAFY* intron II regions were amplified and sequenced following the work of Li et al. [2]. The amplified products of *LEAFY* intron II were purified using the EZNA Cycle-Pure Kit (Omega Bio-Tek, Georgia, USA) before cloning. Cloning was performed using the pEASY-T3 Cloning Kit (TransGen Biotech, Beijing, China). At least 6 positive clones from each individual sample were sequenced and up to 12 positive clones were sequenced for some samples. The sequence chromatogram output files were assembled and edited using Sequencer 4.5 (GeneCodes, Ann Arbor, Michigan, USA).

### Sequence alignment and phylogenetic analyses

DNA sequences were aligned by the program Clustal X 1.81 [20] and edited manually using BioEdit 7.0.9.0 [21]. A single representative sequence was chosen randomly from multiple clones of each individual sample, as all clones from the same individual sample invariably formed a single clade in a preliminary analysis. Individual and combined datasets for the two markers were assembled as ITS, *LEAFY* intron II, and ITS + *LEAFY* intron II. Phylogenetic relationships based on the individual and combined datasets were inferred using unweighted maximum parsimony (MP) by the program PAUP*4.0b10 [22], and Bayesian inference (BI) analyses by the program MrBayes 3.1.2 [23–24].

In the MP analyses, a heuristic search was performed with 100 random addition sequence replicates, tree-bisection-reconnection (TBR) branch swapping, collapse of zero length branches, Multrees on and character state changes unordered and equally weighted. Each random addition sequence replicate was allowed to save up to 1000 trees. Bootstrap support values (BS) of the internal nodes were obtained with 100 bootstrap replicates, using the same options as described above.

In the BI analyses, the best-fit model of evolution was chosen for each dataset (ITS and *LEAFY* intron II) by the program Modeltest 3.7 [25–26] based on the Akaike information criterion (AIC). The Markov chain Monte Carlo (MCMC) algorithm was performed for 2,000,000 generations with one cold and three heated chains, starting from random trees and saving one tree each 100 generations. The first 5000 trees (25%) were discarded as burn-in after checking for stability on the log-likelihood curves, and the remaining 15,000 trees were used to construct the consensus tree. The branch support was determined as Bayesian Posterior Probabilities (BPP).

## Results

### Comparison of morphological characters

**Fig 1:**
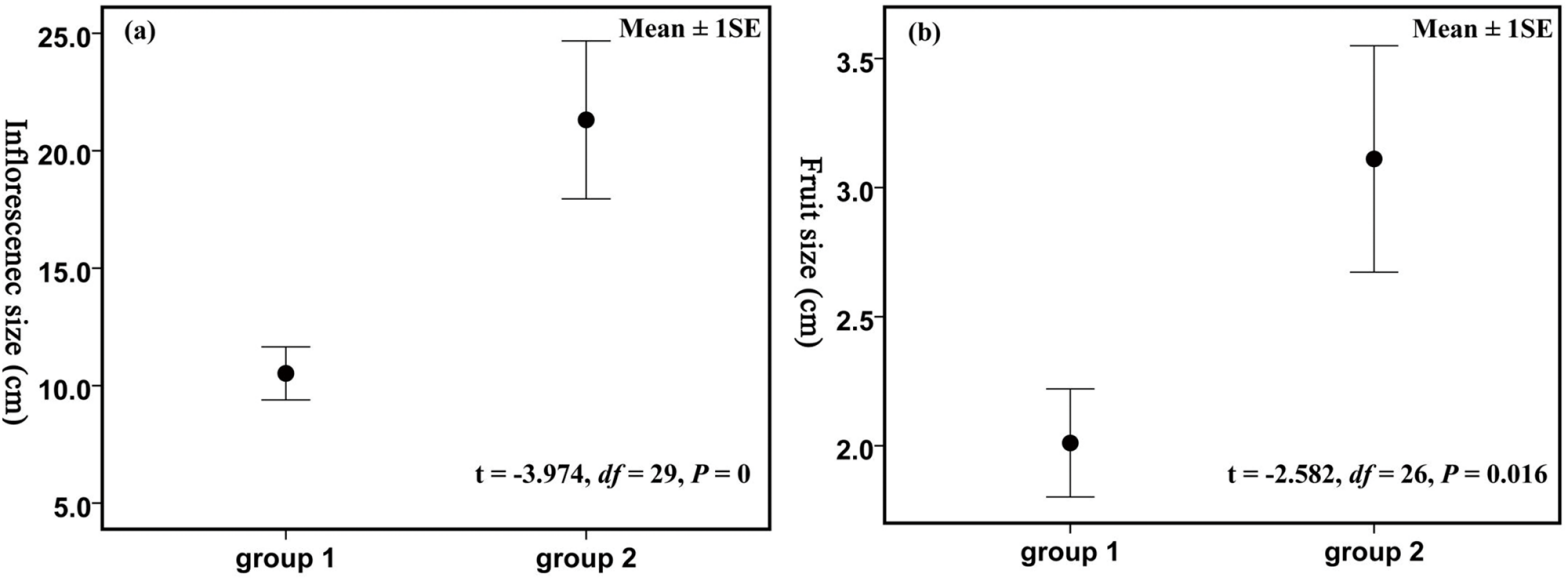
Error bar charts of the inflorescence size and fruit size between group 1 and group 2.

A comparison of morphological characters among the different species of *Alseodaphne* revealed two groups, consisting of species mainly distributed in tropical Asia (group 1) and species mainly distributed in the northern marginal zone of the tropics in southwestern China (group 2), respectively. Group 1 includes the type species, *A*. *semecarpifolia*, whereas the new taxon examined here is placed in group 2. The morphological differences between group 1 species and group 2 species are listed in Table 1. Most of these characters are quantitative and cannot distinguish these two group if considered separately, but in combination, they are effectively separating them. Among the more reliable differences are deciduous perianth lobes in young fruit, not perulate terminal buds as well as whitish twigs contrasting with blackish petioles in dried specimens in group 1 vs. persistent perianth lobes in young fruit, perulate terminal buds, not obviously whitish twigs in group 2. In addition, the error bar charts of the inflorescence size and fruit size between group 1 species and group 2 species showed that both the them were significant (p = 0 and p = 0.016 respectively) (Fig 1). The data of inflorescence size and fruit size are listed in S2 and S3 Tables, respectively.

**Table 1:** Morphological differences between group 1 and group 2.

	Group 1	Group 2
Petiole	Thin, 1–1.5 mm	Thick, 2-4mm
Twig	Thin, 2.5–4.5 mm; obviously whitish in color	Thick, 4–11 mm; not obviously not whitish in color
Terminal bud	Not or rarely perulate	Usually perulate, rarely not perulate
Leaf texture	Variable (thinly chartaceous, chartaceous, thinly coriaceous or coriaceous)	Usually coriaceous, rarely chartaceous
Midrib upper surface	Raised or sunken	Usually sunken, sometimes flat
Inflorescences	Relatively small, 3–20 cm long; few-branched, 1–2 orders; few-flowered	Relatively large, 8.5–35 cm long; many-branched, 3–4 orders; many-flowered
Perianth lobes	Deciduous already in young fruit	± Persistent at least in young fruit
Fruit	Small to medium size, 0.7–3.5.cm; some with ribs	Medium to big size, (1.3) 3–5 cm; without ribs

### Phylogenetic analyses

The two DNA loci, ITS and *LEAFY* intron II, included 600 and 769 aligned position respectively. Modeltest suggested that their evolution was best explained by the TVM + I + G and HKY + G evolutionary models, respectively. The topologies of the consensus trees obtained from the MP and Bayesian analyses, based on different datasets (ITS, *LEAFY* intron II and ITS + *LEAFY* intron II), were mostly congruent. Most of their major clades were identical, and only minor variation in the composition and relationships of a few terminal nodes were detected, possibly caused by insufficient phylogenetic signal in the data and insufficient sample size. Moreover, these inconsistencies received only very weak support. Here, only Bayesian consensus trees with bootstrap support (BS) values and posterior probability support (PPS) values are presented for demonstration.

**Fig 2:**
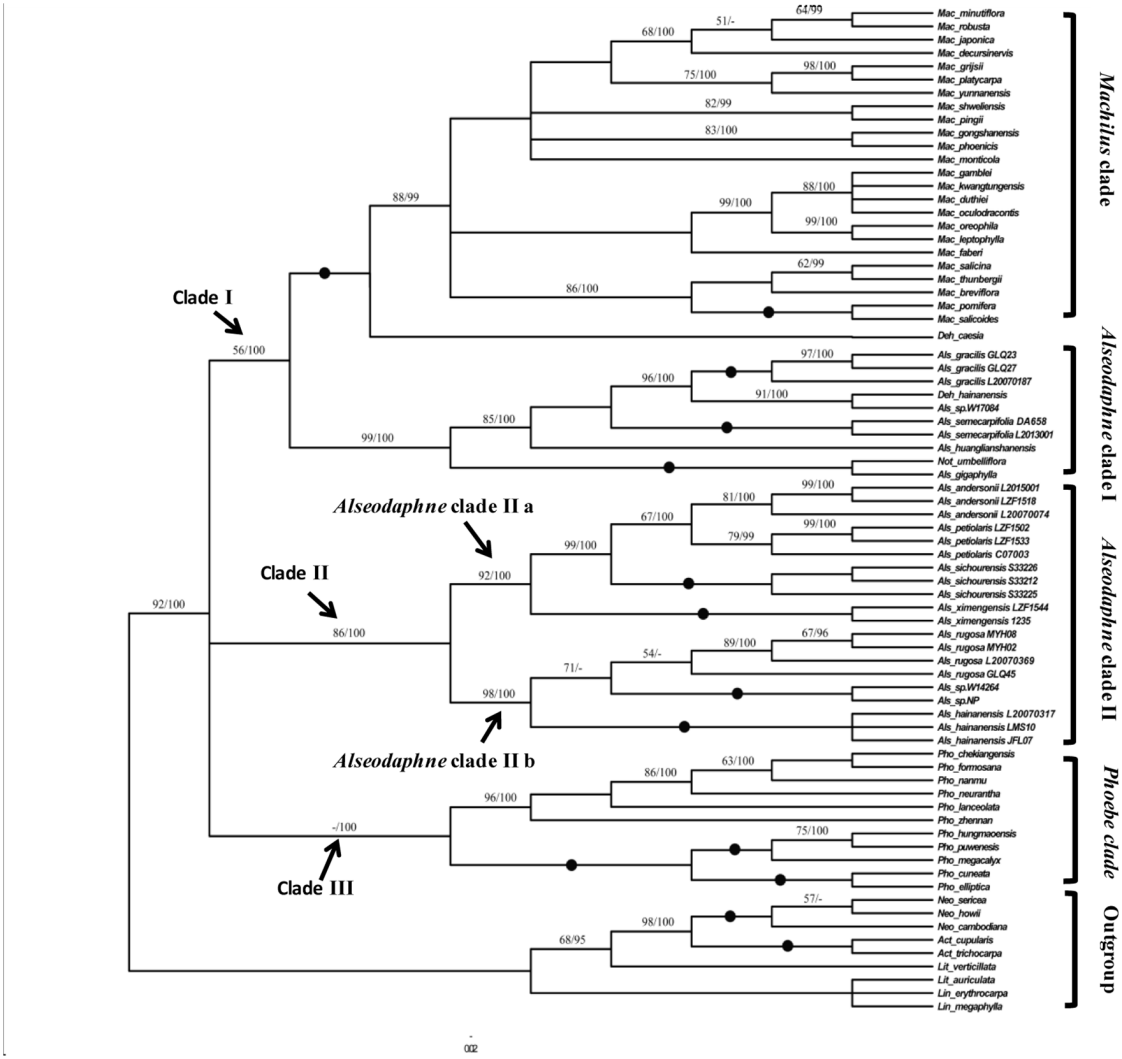
Bayesian consensus tree based on ITS + *LEAFY* intron II combined sequence dataset. Bootstrap values (≥ 50%) / Bayesian posterior probabilities (≥ 95%) are shown above branches. ● = both bootstrap value and Bayesian posterior probability 100%.

The Bayesian consensus tree obtained from the ITS dataset (S1 Fig) is largely congruent with the work of Li et al. [2]. The two principal clades within the *Persea* group are (1) the *Machilus* clade, and (2) a clade including species of *Alseodaphne*, *Dehaasia*, *Nothaphoebe*, and *Phoebe*. Some major clades in the ITS tree, which also appear in the ITS + *LEAFY* intron II tree (Fig 2), are labeled for comparison.

The Bayesian consensus tree obtained from the *LEAFY* intron II dataset (S2 Fig) is also largely compatible with the work of Li et al. [2]. The three principal clades within the *Persea* group are (1) *Alseodaphne* clade II a, (2) *Alseodaphne* clade II b+ *Phoebe* clade, and (3) the *Machilus* clade + a clade including *Alseodaphne*, *Dehaasia*, *Nothaphoebe*. As in S1 Fig, some major clades are labeled for comparison with the ITS + *LEAFY* intron II tree.

The Bayesian consensus tree obtained from the ITS+*LEAFY* intron II dataset (Fig 2) is largely congruent with phylogenies inferred from separate datasets but more resolved and better supported internally. Thus, the ITS + *LEAFY* intron II tree is used for the following discussion and its topology is described below.

Within the ITS + *LEAFY* intron II tree, all species (so far investigated) from the *Persea* group form a well-defined monophyletic clade (92% BS and 100% PPS). We defined three principal clades (clade I, II, and III) within the *Persea* group. Clade I consists of all *Machilus* and *Dehaasia* species included in the present study, and four *Alseodaphne* species distributed in tropical Asia (*A*. *semecarpifolia* and *A*. *gigaphylla*) and southwestern China (*A*. *huanglianshanensis*, *A*. *gracilis*), as well as *Nothaphoebe umbelliflora*. It receives 56% BS and 100% PPS, and has two principal subclades. The major component of the first principal subclade is the *Machilus* Clade, which comprises all representatives of *Machilus* included in the present study. It receives 88% BS and 99% PPS, and shows very little internal resolution. The second principal subclade is *Alseodaphne* cladeⅠ, which consists of four *Alseodaphne* species, *Dehaasia hainanensis*, and *Nothaphoebe umbelliflora*. The *Alseodaphne* clade I receives 99% BS and 100% PPS, and shows very good internal resolution. Clade II consists of *Alseodaphne* species distributed mainly in southwestern China, viz., *A*. *andersonii*, *A*. *hainanensis*, *A*. *petiolaris*, *A*. *rugosa*, *A*. *sichourensis* and the new species described below, plus two unidentified samples (*Alseodaphne sp*. *NP*, *Alseodaphne sp*. *W14264*) that also may represent new species. It receives 86% BS, 100% PPS and has two principal subclades, identical with the *Alseodaphne* clades II a and II b retrieved from the *LEAFY* intron II analysis. Clade III consist of all *Phoebe* species in this study and received 100% PPS but no significant BS. Its two principal subclades, however, are strongly supported in the MP as well as in the BI analyses.

## Discussion

*Machilus* clade and *Phoebe* clade—These two clades have been retrieved in almost identical composition and topology in an earlier analysis [2], so that there is no need to discuss them here again.

*Alseodaphne* clade I and *Alseodaphne* clade II—Just as in the work of Li et al. [2], *Alseodaphne* appears polyphyletic within the *Persea* group, with at least two different origins. Of all the *Alseodaphne* species investigated, four species (*A*. *gracilis*, *A*. *huanglianshanensis*, *A*. *semecarpifolia* and *A*. *gigaphylla*) and one unidentified sample (*Alseodaphne sp*. *W17084*) fall into *Alseodaphne* clade I, whereas six species (*A*. *andersonii*, *A*. *petiolaris*, *A*. *sichourensis*, *A*. *rugosa*, *A*. *hainanensis*, and the new taxon described below) and two unidentified samples (*Alseodaphne sp*. *NP*, *Alseodaphne sp*. *W14264*) are found in an independent clade (*Alseodaphne* clade II). The *Alseodaphne* clade I includes the type species, *A*. *semecarpifolia*, which is most dry-resistant species in *Alseodaphne* [16], so that the name *Alseodaphne* will stay with this clade, which represents the traditionally recognized typical *Alseodaphne* species distributed mainly in tropical Asia.

The origin of the *Alseodaphne* clade II species is apparently different. Most of them are from southwestern China, and *Alseodaphne sp*. *W14264* was collected in northern Vietnam, not far from the Chinese border. The earliest fossil of *Alseodaphne* was found in Changchang Basin of Hainan Island (China), and the extinct *Alseodaphne changchangensis* is closest to the living species *A*. *hainanensis* [27]. Among the extant species, *A*. *hainanensis* and *A*. *rugosa* occur in Hainan, and both are members of *Alseodaphne* clade II. *Alseodaphne hainanensis* also has been reported from northern Vietnam [7]. Hainan Island belongs to the same phytogeographical region as tropical southern China, and it may have been connected to northern Vietnam and Guangxi at least in the Eocene [28]. We also find that the morphological characters within the independent *Alseodaphne* clade II are mainly consistent with the molecular results. This clade is divided into two subclades receiving 92% BS, 100% PPS and 98% BS, 100% PPS respectively (Fig 2). In most of the species of *Alseodaphne* clade II b the lower surfaces of the leaves are distinctly glaucous (except *Alseodaphne sp*. *NP*), while all of the species of *Alseodaphne* clade II a have completely green lower leaf surfaces.

The fact that this independent *Alseodaphne* clade II differs also morphologically from the traditionally recognized *Alseodaphne* species has already been discussed by Rohwer et al. [1]. In this study, we also find some differences between these two clades (Table 1). We therefore think that the independent *Alseodaphne* clade II should be recognized as a new genus, which we call *Alseodaphnopsis*. A formal description of the new genus is provided below. The vegetative characters may be insufficient to distinguish the two genera independently, as most of them are quantitative characters, but in combination, they can be used to segregate the two genera. The principal characters to distinguish the two genera include: 1) twigs thick, 4–11 mm in diameter, not obviously whitish in color vs. thin, 2.5–4.5 mm in diameter, and obviously whitish in color; 2) terminal buds perulate vs. not perulate; 3) perianth lobes persistent at least in young fruit vs. early deciduous; 4) inflorescences relatively large, 8.5–35 cm long, generally many-flowered, with 3–4 order of branching vs. 3–20 cm long, few-flowered, with 1–2 orders of branching; and 5) mature fruit relatively large, 3–5 cm or < 2.5cm in diameter. We found that twigs are not obviously whitish in color and the terminal buds are perulate in almost all species of the new genus *Alseodaphnopsis*, whereas the twigs are obviously whitish in color and terminal buds are not perulate in most of the traditional *Alseodaphne* species. This may be an adaptation to the more seasonal climate in the area of distribution of *Alseodaphnopsis*, with colder winters than in the area of distribution of most *Alseodaphne* species. In addition, the perianth lobes are persistent in young fruit period in at least some species of *Alseodaphnopsis*. We have observed this character in *A*. *andersonii*, *A*. *petiolaris* and *A*. *hainanensis*, but not in the type species *A*. *semecarpifolia*, possibly also caused by the difference of climate. Although the size of panicles and fruits look as quantitative ones, perulate terminal buds as well as persistent perianth lobes look as more or less qualitative ones, these qualitative characters are better to be called as the key characters to define *Alseodaphne* clade II. *Alseodaphne gracilis* and *A*. *huanglianshanensis*, which are distributed in northern tropical Asia, are nested in *Alseodaphne* clade I, not in *Alseodaphne* clade II, as most of the other species from tropical China. Also morphologically, they are more similar to *A*. *semecarpifolia*, which is likewise located in *Alseodaphne* clade I, than to most other Chinese species. We have observed that *A*. *gracilis* has no perianth lobes in its young fruit, which matches with its placement in traditional *Alseodaphne*. In addition, *A*. *gracilis*, *A*. *huanglianshanensis* and *A*. *semecarpifolia* possess thinly leathery leaves and thin twigs with conspicuously whitish bark, whereas the *Alseodaphnopsis* species have leathery leaves and thick twigs that are green or brown, but not whitish in fresh material.

Based on both morphological and molecular evidence, we therefore propose a new genus *Alseodaphnopsis*, separated from the traditional genus *Alseodaphne*, to accommodate the independent *Alseodaphne* clade II including most of the species distributed in the northern margin of tropics in southwestern China (including the new species, *Alseodaphnopsis ximengensis*). In Yunnan (China), at least two further *Alseodaphne* species (*A*. *hokouensis* H. W. Li, *A*. *marlipoensis* (H. W. Li) H. W. Li) belongs to this clade, judged from morphology, so that we can expect this group to become larger with increasing taxon sampling.

### Taxonomic treatment

#### 1. New genus

**Alseodaphnopsis** H. W. Li & J. Li, **gen. nov**. [urn:lsid:ipni.org:names:77165677–1] Type: *Alseodaphnopsis petiolaris* (Meisn.) H. W. Li & J. Li (*Nothaphoebe petiolaris* Meisn., here designated)

**Diagnosis**: The new genus *Alseodaphnopsis* H. W. Li et J. Li is obviously very close to the genus *Alseodaphne* Nees (s. str.), but differs from the latter morphologically by 1) twigs thick, 4–11 mm in diam., and not obviously whitish in color; 2) terminal buds usually perulate; 3) perianth lobes ± persistent at least in young fruit; 4) inflorescences relatively large, 8.5–35 cm long, many-flowered, with 3–4 orders of branching; 5) fruits medium to large size (3–5 cm), without ribs.

**Description**: Trees evergreen. Terminal buds perulate. Twigs robust, 4–10 mm in diam., not whitish. Leaves alternate, pinninerved, always clustered at the ends of the branchlets, abaxial side glaucous or not. Inflorescence axillary, paniculate, bracts and bracteoles deciduous. Flowers bisexual, trimerous. Receptacle short; perianth lobes 6, subequal or extremely unequal, slightly dilated after anthesis and ± persistent at least in young fruit. Fertile stamens 9, in 3 whorls; filaments of 1st and 2nd whorls glandless, those of 3rd whorl each with 2 glands at base; anthers 4-locular; locules of 1st and 2nd whorls introrse, those of 3rd whorl extrorse or upper locules lateral and lower extrorse. Staminodes 3, of innermost whorl, small, clavate to nearly sagittate. Ovary partly immersed into shallow receptacle; style shorter than ovary; stigma discoid. Fruit medium to big size, 3–5 cm in diam., without ribs, black or purplish black when mature, oblong or subglobose; fruit stalk slightly enlarged or much enlarged, red, green, or yellow, nearly cylindric or obconical, fleshy or somewhat woody, always warty.

**Etymology**: *Alseodaphnopsis* alludes to the resemblance to traditional *Alseodaphne* (s. str.)

**Distribution and habitat**: *Alseodaphnopsis* includes nine species, mainly distributed in the northern marginal part of the tropical zone in southwestern China, but extending also to NE India, Laos, Myanmar, Thailand and Vietnam. As far as it is known, the species grow preferentially in forests on limestone mountains.

#### 2. New combinations

Here, we make eight new combinations for the species in this new genus as follows:

**1) *Alseodaphnopsis andersonii*** (King ex Hook. f.) H. W. Li & J. Li, **comb. nov**. [urn:lsid:ipni.org:names:77165678–1] Type: Assam, fl., Jenkins *s*.*n*, (BO!, CAL, K)

Basionym: *Cryptocarya andersonii* King ex Hook. f., Fl. Brit. India. 5: 120. 1886 ≡ *A*. *andersonii* (King ex Hook. f.) Kosterm., Reinwardtia 6 (2): 159. 1962.

= *Alseodaphne keenanii* Gamble, Kew Bull. 1914: 188.

Distributed in China (SE & S Yunnan, SE Xizang); NE India, Laos, Myanmar, Thailand and Vietnam.

**2) *Alseodaphnopsis petiolaris*** (Meisn.) H. W. Li & J. Li, **comb. nov**. [urn:lsid:ipni.org:names:77165679–1] Type: Assam, Nuka Hills, Simons *s*.*n*. (BO!, CAL, K)

Basionym: *Nothaphoebe petiolaris* Meisn. in A. Candolle, Prodr. 15(1): 59. 1864.

≡ *Alseodaphne petiolaris* (Meisn.) Hook. f., Fl. Brit. India 5: 145. 1886; *Persea petiolaris* (Meisn.) Debarman, Bull. Bot. Surv. India 32: 257. 1962.

Distributed in China (S Yunnan); India and Myanmar.

**3) *Alseodaphnopsis sichourensis*** (H. W. Li) H. W. Li & J. Li, **comb. nov**. [urn:lsid:ipni.org:names:77165680–1] Type: China. Yunnan: Sichour, Tsaokuoshan, Wen-shan Exp. 61–077 (KUN!)

Basionym: *Alseodaphne sichourensis* H. W. Li, Act. Phytotax. Sin. 17 (2): 70. 1979.

Distributed in China (SE Yunnan).

**4) *Alseodaphnopsis marlipoensis*** (H. W. Li) H. W. Li & J. Li, **comb. nov**. [urn:lsid:ipni.org:names:77165681–1] Type: China. Yunnan: Marlipo, Tianbao Farm, S.C. Wang 81 (KUN!)

Basionym: *Cinnamomum marlipoensis* H. W. Li, Act. Phytotax. Sin. 13 (4): 48. 1975.

≡ *Alseodaphne marlipoensis* (H. W. Li) H. W. Li, Act. Phytotax. Sin. 17 (2): 71. 1979.

Distributed in China (SE Yunnan).

**5) *Alseodaphnopsis rugosa*** (Merr. & Chun) H. W. Li & J. Li, **comb. nov**. [urn:lsid:ipni.org:names:77165682–1] Type: China. Hainan: Chun & Tso 44254 (A!)

Basionym: *Alseodaphne rugosa* Merr. & Chun, Sunyatsentia 2: 232. 1935.

Distributed in China (Hainan, SE Yunnan).

**6) *Alseodaphnopsis hainanensi*s** (Merr.) H. W. Li & J. Li, **comb. nov**. [urn:lsid:ipni.org:names:77165683–1] Type: China. Hainan: Tsang & Fung 766 = L.U.18300 (A!, BO!, DD, K, L)

Basionym: *Alseodaphne hainanensis* Merr., Lingnan Sci. J. 13: 57. 1934.

Distributed in China (Hainan); N Vietnam.

**7) *Alseodaphnopsis hokouensis*** (H. W. Li) H. W. Li & J. Li, **comb. nov**. [urn:lsid:ipni.org:names:77165684–1] Type: China. Yunnan: Hokou, K.H. Tsai 1039 (KUN!)

Basionym: *Alseodaphne hokouensis* H. W. Li, Act Phytotax. Sin. 17 (2): 71. 1979.

Distributed in China (SE Yunnan).

8) ***Alseodaphnopsis lanuginosa*** (Kosterm.) H. W. Li & J. Li, **comb. nov**. [urn:lsid:ipni.org:names:77165685–1] Type: Vietnam. Tonkin: Chapa, Petelot 3565(BO!, P)

Basionym: *Alseodaphne lanuginosa* Kosterm., Candollea 28: 116. 1973.

Distributed in N Vietnam.

Note: Four additional species from SW China and N Vietnam, *A*. *hokouensis*, *A*. *yunnanensis*, *A*. *marlipoensis* and *A*. *lanuginosa* are not contained in our phylogenetic analyses, but according to the morphological characters, *A*. *hokouensis*, *A*. *marlipoensis* and *A*. *lanuginosa* are similar to *A*. *petiolaris*, *A*. *sichourensis* and *A*. *andersonii*, which belong to *Alseodaphnopsis* [16, 29], while *A*. *yunnanensis* is similar to *A*. *huanglianshanensis* which belongs to *Alseodaphne* [30]. We therefore treat *A*. *hokouensis*, *A*. *marlipoensis* and *A*. *lanuginosa* in *Alseodaphnopsis* while *A*. *yunnanensis* is retained in *Alseodaphne*.

#### 3. New species

**Fig 3:**
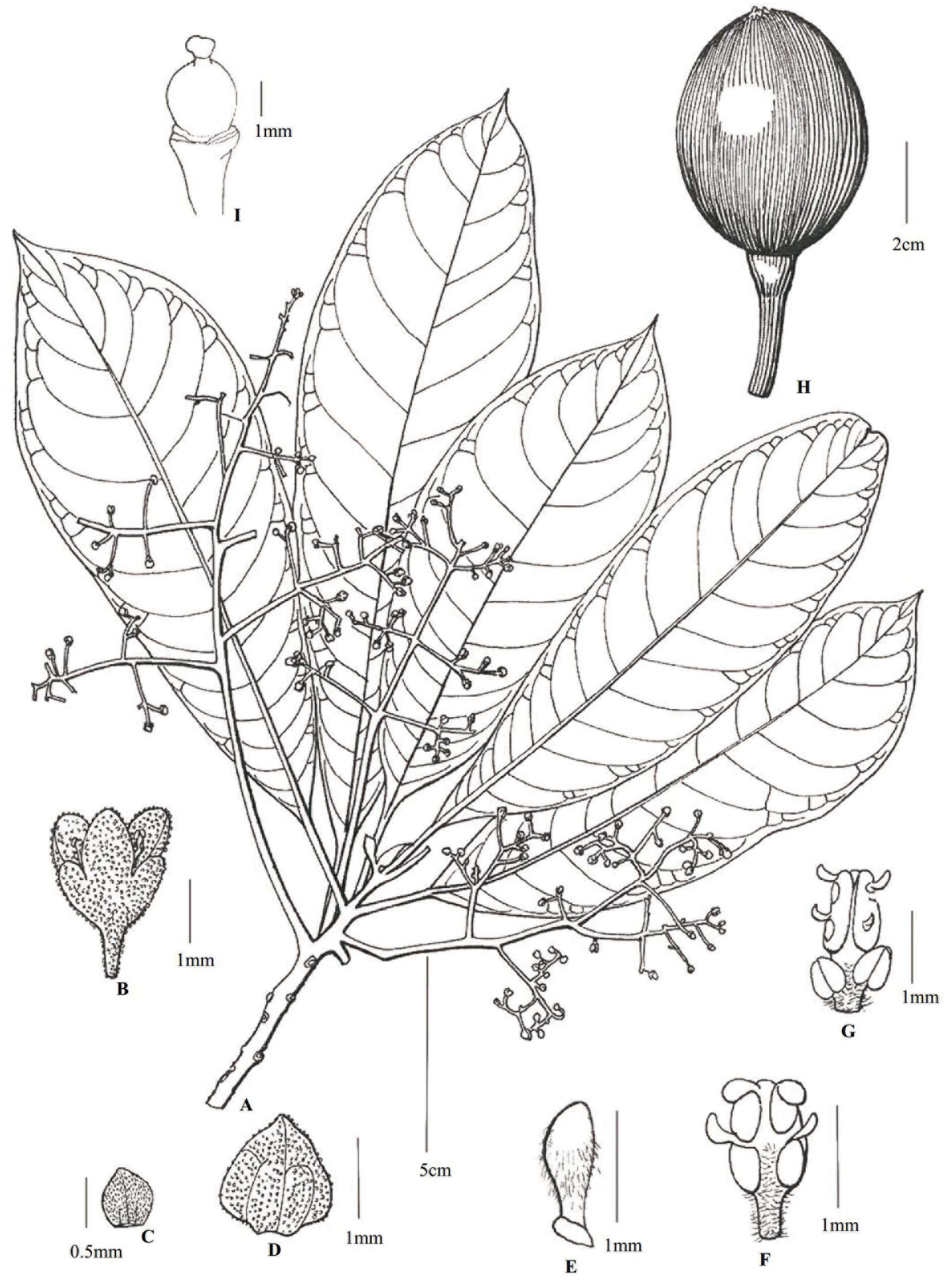
*Alseodaphnopsis ximengensis* H. W. Li & J. Li *sp*. *nov*. A. Flowering branch; B. Flower, lateral view; C. Outer perianth lobes, outside view; D. Inner perianth lobes, inside view; E. A staminode; F. A fertile stamen of the 1st or 2nd whorl; G. A stamen of the third whorl; H. Fruit; I. Pistil. (drawn by L. Wang based on J. W. Li 1235 sampled from Ximeng County, Yunnan).

**Fig 4:**
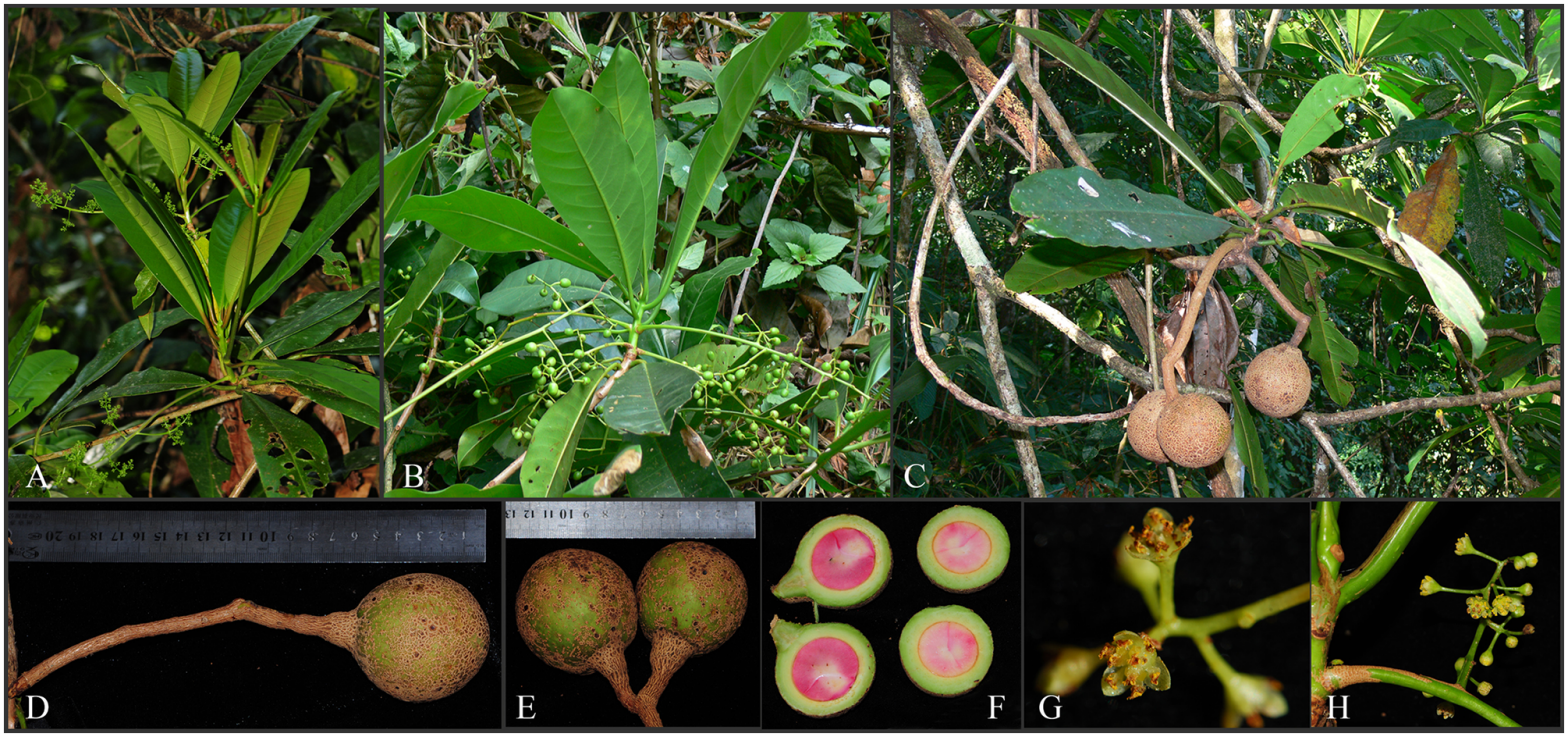
*Alseodaphnopsis ximengensis* H. W. Li & J. Li *sp*. *nov*. A. Branchlet with inflorescences; B. Branchlet with immature infructescences; C. Branchlet with mature fruits; D-F. Mature fruits; G-H. Flowers. (Photos by J.W. Li).

**Alseodaphnopsis ximengensis** H. W. Li & J. Li, **sp. nov**. (Figs 3 and 4) [urn:lsid:ipni.org:names:77165686–1] Type: China. Yunnan Province: Pu’er City, Ximeng County, ca. 1300 m altitude, 22°41′28.36″N, 99°38′59.68″E, in seasonal rain forest, 20 November 2011, *J*. *W*. *Li 1235* (fl.) (Holotype, Isotypes: HITBC!).

**Diagnosis**: This new species shows a superficial similarity to *Alseodaphnopsis petiolaris* (Meisn.) H. W. Li & J. Li in its big leaves and elongated petioles, but differs by its glabrous twigs, leaves and panicles as well as subglobose big fruit.

**Description**: Trees evergreen. One-year-old branchlets robust, 8–11 mm in diam., yellowish-brown, glabrous, with elevated orbicular lenticels and large suborbicular leaf scars; current year branchlets slender, elongate, terete, 4–6 mm in diam., glabrous, green when young but all brown when dry. Terminal buds large, ca.1 cm, glabrous. Leaves clustered at apex of branchlet; petiole 2–4 mm thick, 2.5–4 cm long, concave-convex; leaf blade greenish on both surfaces, red-brown when young, oblong-oblanceolate, 17–30 × 6–11 cm, leathery, glabrous on both surfaces, midrib conspicuously elevated abaxially, impressed adaxially, lateral veins 13–17 pairs, elevated on both surfaces, arcuately connected at ends, base cuneate, apex acute to obtuse with a short acumen of 5–7 mm. Panicle axillary, glabrous, 20–30 cm long, with 6–14 lateral branches and 3–4 orders of branching; peduncle 3–7.5 cm. Flowers small, ca. 2 mm long; pedicels slender, 2–4 mm, dilated in fruit. Perianth lobes 6, broadly ovate, white pubescent on margin; outer ones smaller, ca. 0.5×0.5 mm, inner ones larger, ca. 1.5×1.5 mm, deciduous in mature fruit. Fertile stamens 9, ca. 1.5 mm in 1st whorl, ca. 1.7 mm in 2nd whorl, ca. 2 mm in 3rd whorl; filaments villous, very short, those of 3rd whorl each with 2 stalkless glands at base, others glandless; anthers of 1st and 2nd whorls elliptic, with 2 upper slightly smaller locules and 2 lower large locules, locules all introrse, anthers of 3rd whorl oblong, with 2 upper smaller locules and 2 lower large locules, locules all latrorse-extrorse. Ovary globose, ca. 2 mm long, glabrous, style short; stigma conspicuous. Fruit large, subglobose, green when young but brown or black when dry, ca. 4.7 cm in diam.; fruit stalk robust, 9–16 (29) cm long, dilated at the tip, up to 8 mm in diam. Fl. November, fr. July-August of next year.

**Additional specimens examined (paratypes)**: China. Yunnan Province: Pu’er City, Ximeng County, ca. 1300 m altitude, in seasonal rain forest, 25 January 2011, *J*. *W*. *Li 283* (fr.) (HITBC!, same tree with the holotype *J*. *W*. *Li 1235*); China. Yunnan Province: Pu’er City, Ximeng County, 10 November 1985, collector: Y. Y. Qian (No collecting number) (HITBC!, HITBC No. 110940, (fl.)).

## Supporting information

S1 Table Voucher information and GenBenk accessions for ITS and LFY sequences for species examined in this study. Sequences starting with AY, FM, FJ, and HQ come from work of Li et al. (unpublished), Rohwer et al. (2009), Chen et al (2009), and Li et al. (2011) respectively.
(DOCX)

S2 Table Size of inflorescence. The data of inflorescence are gathered in the relevant literatures below the table, except for *A*. *ximengensis* H. W. Li et J. Li and *A*. *sp*. *NP*.
(DOCX)

S3 Table Shape and size (diam.) of fruit. The data of fruit are gathered in the relevant literatures below the table, except for *A*. *ximengensis* H. W. Li et J. Li and *A*. *sp*. *NP*.
(DOCX)

S1 Fig Bayesian consensus tree based on ITS sequence dataset. Bootstrap values (≥ 50%) / Bayesian posterior probabilities (≥ 95%) are shown above branches. ● = both bootstrap value and Bayesian posterior probability 100%.
(PDF)

S2 Fig Bayesian consensus tree based on *LEAFY* intron II sequence dataset. Bootstrap values (≥ 50%) / Bayesian posterior probabilities (≥ 95%) are shown above branches. ● = both bootstrap value and Bayesian posterior probability 100%.
(PDF)
