# Supplementary material for: Alseodaphnopsis: A new genus of Lauraceae based on molecular and morphological evidence
Source: PLoS One. 2017 Oct 18;12(10):e0186545. doi: 10.1371/journal.pone.0186545 (PMC5646853; doi:10.1371/journal.pone.0186545)
Supplement: S1 Table — Sequences starting with AY, FM, FJ, and HQ come from work of Li et al. (unpublished), Rohwer et al. (2009), Chen et al (2009), and Li et al. (2011) respectively. (DOCX) [file pone.0186545.s001.docx]

**S1 Table. Voucher information and GenBenk accessions for ITS and LFY sequences for species examined in this study.** The sequences starting with MG are new from present study; sequences starting with AY, FM, FJ, and HQ come from work of Li et al. (unpublished), Rohwer et al. (2009), Chen et al (2009), and Li et al. (2011) respectively.

| **Taxon** | **Voucher** | **Locality** | **ITS** | **LFY** |
| --- | --- | --- | --- | --- |
| **Ingroups** |  |  |  |  |
| ***Alseodaphne* (13)** |  |  |  |  |
| *Als. andersonii* (King ex Hook. f.) Kosterm. | Li J. & Li L. 20070074 (HITBC) | China, Yunnan | FM957793 | HQ697002 |
|  | Li J. et al. LZF1518 (HITBC) | China, Yunnan | MG188593 | MG188612 |
|  | L S.Y. 2015001 (HITBC) | China, Yunnan | MG188592 | MG188606 |
| *Als. gigaphylla* Kosterm. | Arifiani DA657 (BO) | Indonesia, Java | HQ697181 | HQ697004 |
| *Als. gracilis* Kosterm. | Li L. 20070187 (HITBC) | China, Yunnan | HQ697187 | HQ697036 |
|  | Li L.et al. GLQ23 (HITBC) | China, Yunnan | MG188581 | MG188659 |
|  | Li L.et al. GLQ27 (HITBC) | China, Yunnan | MG188582 | MG188655 |
| *Als. hainanensis* Merr. | Li L. & Wang Z. H. 20070317 (HITBC) | China, Hainan | FJ755440 | HQ697005 |
|  | Li L. & Wang Z. H. JFL07 (HITBC) | China, Hainan | MG188587 | MG188634 |
|  | Li L. & Wang Z. H. LMS10 (HITBC) | China, Hainan | MG188586 | MG188633 |
| *Als. huanglianshanensis* H. W. Li &Y. M. Shui | Li L. 20080006 (HITBC) | China, Yunnan | HQ6971812 | HQ697007 |
| *Als. petiolaris* (Meissn.) Hook. f. | Chen J. Q. 07003 (HITBC) | China, Yunnan | FM957796 | HQ697008 |
|  | Li J. et al. LZF 1502 (HITBC) | China, Yunnan | MG188594 | MG188618 |
|  | Li J. et al. LZF1533 (HITBC) | China, Yunnan | MG188595 | MG188619 |
| *Als. rugosa* Merr. & Chun | Li L. & Wang Z. H. MYH08 (HITBC) | China, Hainan | MG188584 | MG188640 |
|  | Li L. & Wang Z. H. MYH02 (HITBC) | China, Hainan | MG188585 | MG188635 |
|  | Li L. & Wang Z. H. 20070369 (HITBC) | China, Hainan | HQ6971813 | HQ697011 |
|  | Li L. et al. GLQ45 (HITBC) | China, Yunnan | MG188588 | MG188646 |
| *Als. semecarpifolia* Nees | Arifiani DA658 (BO) | Indonesia, Java | HQ6971814 | HQ697015 |
|  | Li L. 2013001 (BO) | Indonesia, Java | MG188583 | MG188665 |
| *Als. sichourensis* H. W. Li | Song Y. 33225 (HITBC) | China, Yunnan | MG188597 | MG188626 |
|  | Song Y. 33226 (HITBC) | China, Yunnan | MG188596 | MG188627 |
|  | Song Y. 33212 (HITBC) | China, Yunnan | MG188598 | MG188620 |
| *Als.* sp. W14264 | van der Werff & Nguyen 14264 (MO) | Vietnam, Tuyen Quang | FM957797 | HQ697016 |
| *Als.* sp. W17084 | van der Werff et al., 17084 (MO) | Vietnam, Lang Son | FM957798 | HQ697020 |
| *Als. s*p. NP | Li L.et al.20160030 | China, Guangxi | MG188589 | MG188654 |
| *Als. ximengensis* sp. nov. | Li J. et al. LZF1544 | China, Yunnan | MG188590 | MG188600 |
|  | Li J. W. 1235 | China, Yunnan | MG188591 | MG188599 |
| ***Dehaasia* (2)** |  |  |  |  |
| *Deh. caesia* Blume | Arifiani DA493 (BO) | Indonesia, Java | HQ697185 | HQ697024 |
| *Deh. hainanensis* Kosterm. | Li L. & Wang Z. H. 20070373 (HITBC) | China, Hainan | FJ719308 | HQ697026 |
| ***Machilus* (24)** |  |  |  |  |
| *Mac. breviflora* (Benth.) Hemsl. | Chen J. Q. et al. 2006013 (HITBC) | China, Guangdong | FJ755434 | HQ697041 |
| *Mac. decursinervis* Chun | Li J. 2002195 (HITBC) | China, Guangxi | AY934893 | HQ697044 |
| *Mac. duthiei* King ex Hook. f. | Zhong J. S. 2006094 (HITBC) | China, Yunnan | FJ755425 | HQ697055 |
| *Mac. faberi* (Hemsl.) Chun | Li L. 20070269 (HITBC) | China, Sichuan | HQ697204 | HQ697133 |
| *Mac. gamblei* King ex Hook. f. | Chen J. Q. et al. 2006001 (HITBC) | China, Guangdong | FJ755422 | HQ697040 |
| *Mac. gongshanensis* H. W. Li | Chen J. Q. 07002 (HITBC) | China, Yunnan | FJ755416 | HQ697047 |
| *Mac. grijsii* Hance | Chen J. Q. et al. 2006028 (HITBC) | China, Guangdong | FJ755420 | HQ697049 |
| *Mac. japonica* Sieb. & Zucc. | Kim C. K. s.n. (HBG) | Korea, Cheju | AY934891 | HQ697050 |
| *Mac. kwangtungensis* Yang | Chen J. Q. et al. 2006027 (HITBC) | China, Guangdong | FJ755424 | HQ697051 |
| *Mac. leptophylla* Hand-Mazz. | Li J. & Li L. 20070190 (HITBC) | China, Zhejiang | FJ755430 | HQ697053 |
| *Mac. minutiflora* H. W. Li | Chen J. Q. et al. 2005038 (HITBC) | China, Yunnan | HQ697208 | HQ697148 |
| *Mac. monticola* S. Lee | Li L. & Wang Z. H. 20070323 (HITBC) | China, Hainan | FJ755418 | HQ697057 |
| *Mac. oculodracontis* Chun | Chen J. Q. et al. 2006037 (HITBC) | China, Guangdong | HQ697188 | HQ697061 |
| *Mac. oreophila* Hance | Chen J. Q. et al. 2006067 (HITBC) | China, Guangdong | FJ755423 | HQ697063 |
| *Mac. phoenicis* Dunn | Chen J. Q. et al. 2006009 (HITBC) | China, Guangdong | FJ755413 | HQ697064 |
| *Mac. pingii* Cheng ex Yang | Li L. 20070263 (HITBC) | China, Sichuan | HQ697189 | HQ697065 |
| *Mac. platycarpa* Chun | Chen J. Q. et al. 2006073 (HITBC) | China, Guangdong | FJ755421 | HQ697067 |
| *Mac. pomifera* (Kosterm.) S. Lee | Chen J. Q. et al. 2006064 (HITBC) | China, Guangdong | FJ755432 | HQ697070 |
| *Mac. robusta* W. W. Sm. | Li J. 2002116 (HITBC) | China, Guangxi | FJ755426 | HQ697071 |
| *Mac. salicina* Hance | Chen J. Q. et al. 2005001 (HITBC) | China, Yunnan | FJ755428 | HQ697073 |
| *Mac. salicoides* S. Lee | Chen J. Q. et al. 2006090 (HITBC) | China, Guangdong | FJ755433 | HQ697074 |
| *Mac. shweliensis* W. W. Sm. | Li J. 2002087 (HITBC) | China, Guangxi | FJ755414 | HQ697075 |
| *Mac. thunbergii* Sieb. & Zucc. | Rohwer s.n. (HBG) | Germany, Hamburg | HQ697190 | HQ697082 |
| *Mac. yunnanensis* Lec*.* | Zhong J. S. 2006093 (HITBC) | China, Yunnan | FJ755415 | HQ697084 |
| ***Nothaphoebe* (1)** |  |  |  |  |
| *Not. umbelliflora* (Blume) Blume | Arifiani DA495 (BO) | Indonesia, Java | HQ697191 | HQ697088 |
| ***Phoebe* (10)** |  |  |  |  |
| *Pho. chekiangensis* C. B. Shang | Li J. & Li L. 20070188 (HITBC) | China, Zhejiang | FJ755407 | HQ697128 |
| *Pho. cuneata* (Blume) Blume | Arifiani 40 (MO) | Indonesia | HQ697202 | HQ697130 |
| *Pho. elliptica* (Blume) Blume | Samsuri & Gwee SING2004-28 (SBG) | Singapore | HQ697203 | HQ697131 |
| *Pho. formosana* (Matsum. & Hay.) Hay. | Rohwer 156 (MJG) | Germany, Bonn | HQ697205 | HQ697136 |
| *Pho. hungmaoensis* S. Lee | Li L. & Wang Z. H. 20070306 (HITBC) | China, Hainan | HQ697206 | HQ697140 |
| *Pho. lanceolata* (Wall. ex Nees) Nees | Chen J. Q. et al. 2006093 (HITBC) | China, Guangdong | FJ755410 | HQ697141 |
| *Pho. megacalyx* H. W. Li | Li J. & Li L. 20070026 (HITBC) | China, Yunnan | HQ697207 | HQ697144 |
| *Pho. nanmu* (Oliv.) Gamble | Chen J. Q. et al. 2005002 (HITBC) | China, Yunnan | FJ755409 | HQ697150 |
| *Pho. neurantha* (Hemsl.) Gamble | Li J. & Li L. 20070214 (HITBC) | China, Zhejiang | HQ697209 | HQ697151 |
| *Pho. puwenensis* Cheng | Chen J. Q. et al, 2006065 (HITBC) | China, Guangdong | HQ697210 | HQ697156 |
| **Outgroups** |  |  |  |  |
| ***Actinodaphne* (2)** |  |  |  |  |
| *Act. cupularis* (Hemsl.) Gamble | Li L. 20070231 (HITBC) | China, Sichuan | HQ697213 | HQ697164 |
| *Act. trichocarpa* C. K. Allen | Li L. 20070282 (HITBC) | China, Sichuan | HQ697214 | HQ697166 |
| ***Lindera* (2)** |  |  |  |  |
| *Lin. erythrocarpa* Makino | Li J. & Li L. 20070203 (HITBC) | China, Zhejiang | HQ697215 | HQ697167 |
| *Lin. megaphylla* Hemsl. | Li L. 20070236 (HITBC) | China, Sichuan | HQ697216 | HQ697172 |
| ***Litsea* (2)** |  |  |  |  |
| *Lit. auriculata* Chien et Cheng | Li J. & Li L. 20070195 | China, Zhejiang (HITBC) | HQ697217 | HQ697174 |
| *Lit. verticillata* Hance | Li L. & Wang Z. H. 20070337 (HITBC) | China, Hainan | HQ697218 | HQ697175 |
| ***Neolitsea* (3)** |  |  |  |  |
| *Neo. cambodiana* Lec. | Li L. & Wang Z. H. 20070327 (HITBC) | China, Hainan | HQ697219 | HQ697176 |
| *Neo. howii* C. K. Allen | Li L. & Wang Z. H. 20070379 (HITBC) | China, Hainan | HQ697220 | HQ697178 |
| *Neo. sericea* (Blume) Koidz. | Li J. & Li L. 20070225 (HITBC) | China, Zhejiang | HQ697221 | HQ697180 |
