# Supplementary material for: Alseodaphnopsis: A new genus of Lauraceae based on molecular and morphological evidence
Source: PLoS One. 2017 Oct 18;12(10):e0186545. doi: 10.1371/journal.pone.0186545 (PMC5646853; doi:10.1371/journal.pone.0186545)
Supplement: S2 Table — The data of inflorescence are gathered in the relevant literatures below the table, except for A. ximengensis H. W. Li et J. Li and A. sp. NP. (DOCX) [file pone.0186545.s002.docx]

**S2 Table.** **Size of inflorescence.** The data of inflorescence are gathered in the relevant literatures below the table, except for *A. ximengensis* H. W. Li et J. Li and *A. sp. NP*.

| **Species** | **Size of inflorescence (cm)** | **Group** |
| --- | --- | --- |
| *Alseodaphne albifrons* Kosterm. | up to 8 | group 1 |
| *Alseodaphne bancana* Miq. | 10 | group 1 |
| *Alseodaphne birmanica* Kosterm. | 3-6 | group 1 |
| *Alseodaphne borneensis* Gamble | 11 | group 1 |
| *Alseodaphne corneri* Kosterm. | up to 8 | group 1 |
| *Alseodaphne dura* Kosterm. | up to 5 | group 1 |
| *Alseodaphne elata* Kosterm. | up to 8 | group 1 |
| *Alseodaphne glauciflora* Kosterm. | up to 8 | group 1 |
| *Alseodaphne gigaphylla* Kosterm. | up to 20 | group 1 |
| *Alseodaphne gracilis* Kosterm. | up to 3 | group 1 |
| *Alseodaphne habrotricha* Kosterm. | 12-18 | group 1 |
| *Alseodaphne intermedia* Kosterm. | up to 17 | group 1 |
| *Alseodaphne macrantha* Kosterm. | up to 11 | group 1 |
| *Alseodaphne micrantha* Kosterm. | 1 | group 1 |
| *Alseodaphne montana* Kosterm. | 6-15 | group 1 |
| *Alseodaphne ramosii* Kosterm. | 3-5 | group 1 |
| *Alseodaphne rhododendropsis* Kosterm. | up to 13 | group 1 |
| *Alseodaphne rubriflora* Kosterm. | 6-14 | group 1 |
| *Alseodaphne semecarpifolia* Nees | 10-20 | group 1 |
| *Alseodaphne siamensis* Kosterm. | 5-16 | group 1 |
| *Alseodaphne suboppositifolia* Kosterm. | 10 | group 1 |
| *Alseodaphne tomentosa* Kosterm. | up to 11 | group 1 |
| *Alseodaphne yunnanensis* Kosterm. | 2-4 | group 1 |
| *Alseodaphne andersonii* (King ex Hook. f.) Kosterm. | 20-35 | group 2 |
| *Alseodaphne hainanensis* Merr. | 3.5-12 | group 2 |
| *Alseodaphne hokouensis* H.W. Li | 10.5-15 | group 2 |
| *Alseodaphne lanuginosa* Kosterm. | up to 20 | group 2 |
| *Alseodaphne petiolaris* Hook. f | 10-30 | group 2 |
| *Alseodaphne rugosa* Merr. & Chun | 15-20 | group 2 |
| *Alseodaphne sichourensis* H.W. Li | 5-8.5 | group 2 |
| *Alseodaphne ximengensis* H.W. Li et J. Li | 20-30 | group 2 |

References

**1.** Li, HW, PAI, PY, LEE, SK, WEI, FN, Yang, YC, Huang, PH, et al. *Lauraceae*. In: Li, HW (Ed.), Flora Reipublicae Popularis Sinicae, vol. 31. Science Press, Beijing, China; 1982.

**2.** Li, HW, Li, J, Huang, PH, Wei, FN & van der Werff, H. *Lauraceae*. In: Wu, ZY, Raven, PH, Hong, DY (Eds.), Flora of China, vol. 7. Science Press and Missouri Botanical Garden Press, Beijing, China, St. Louis, Missouri, USA; 2008.

**3**. Kostermans, AJGH. New and critical Malaysian plants IV. Reinwardtia, 1956; 4, 1-40.

**4.** Kostermans, AJGH. Lauraceae. Reinwardtia, 1957; 4, 275-277.

**5**. Kostermans, AJGH. New and critical Malaysian plants VI. Reinwardtia, 1960; 5, 341-369.

**6**. Kostermans, AJGH. Materials for a revision of Lauraceae I. Reinwardtia, 1968; 7, 291-356.

**7**. Kostermans, AJGH. Materials for a revision of Lauraceae III. Reinwardtia, 1970; 8, 21-196.

**8.** Kostermans, AJGH. A synopsis of *Alseodaphne* Nees (Lauraceae). *Candollea*, 1973; 28, 93-136.

**9**. Mo, YQ, Li, L, Zhang, JG, Li, HW & Li, J. Discovery of *Alseodaphne* *rugosa* Merr. et Chun (Lauraceae) in SE Yunnan, China and its biogeographical significance. Guihaia, 2017; 37, (In Press)

**10**. Thakur, BK, Anthwal, A, Rawat, DS, Rawat, B, Rashmi & Rawat, M. A review on genus *Alseodaphne*: phytochemistry and pharmacology. Mini-Reviews in Organic Chemistry, 2012; 9, 433-445.
