# Supplementary material for: Alseodaphnopsis: A new genus of Lauraceae based on molecular and morphological evidence
Source: PLoS One. 2017 Oct 18;12(10):e0186545. doi: 10.1371/journal.pone.0186545 (PMC5646853; doi:10.1371/journal.pone.0186545)
Supplement: S3 Table — The data of fruit are gathered in the relevant literatures below the table, except for A. ximengensis H. W. Li et J. Li and A. sp. NP. (DOCX) [file pone.0186545.s003.docx]

**S3 Table.** **Shape and size (diam.) of fruit.** The data of fruit are gathered in the relevant literatures below the table, except for *A. ximengensis* H. W. Li et J. Li and *A. sp. NP*.

| **Species** | **Size (diam.) of fruit (cm)** | **Group** |
| --- | --- | --- |
| *Alseodaphne bancana* Miq. | subglobose, 1.3 | group 1 |
| *Alseodaphne birmanica* Kosterm. | ellipsoid, 1.7 | group 1 |
| *Alseodaphne borneensis* Gamble | ellipsoid, 4.5 | group 1 |
| *Alseodaphne corneri* Kosterm. | ellipsoid, 2.0 | group 1 |
| *Alseodaphne elmeri* Merr. | ellipsoid, 1.5 | group 1 |
| *Alseodaphne foxiana* (Gamble) Kosterm. | ellipsoid, 2.2 | group 1 |
| *Alseodaphne garciniaecarpa* Kosterm. | subobovoid, truncated, 2.0 | group 1 |
| *Alseodaphne habrotricha* Kosterm. | ellipsoid, 0.7 | group 1 |
| *Alseodaphne huanglianshanensis* H.W. Li & Y.M. Shui | ovoid, 1.4 | group 1 |
| *Alseodaphne himalayana* Kosterm. | globose, 1.0 | group 1 |
| *Alseodaphne intermedia* Kosterm. | ellipsoid, 1.8 | group 1 |
| *Alseodaphne kochummenii* Kosterm. | globose, 1.7 | group 1 |
| *Alseodaphne montana* Kosterm. | globular, 3.5 | group 1 |
| *Alseodaphne owdeni* R. Parker | ellipsoid, 1.8 | group 1 |
| *Alseodaphne peduncularis* (Wall. ex Nees) | ellipsoid, 2.0 | group 1 |
| *Alseodaphne pendulifolia* Gamble | subglobose, 1.6 | group 1 |
| *Alseodaphne semecarpifolia* Nees | ellipsoid, 1.5 | group 1 |
| *Alseodaphne sulcata* Kosterm. | subovoid-ellipsoid, 3.0 | group 1 |
| *Alseodaphne utilis* Kosterm. | ellipsoid, 3.0 | group 1 |
| *Alseodaphne andersonii* (King ex Hook. f.) Kosterm. | oblong, 2.8 | group 2 |
| *Alseodaphne hainanensis* Merr. | globose or ovoid, 2.0 | group 2 |
| *Alseodaphne lanuginosa* Kosterm. | ellipsoid-ovoid, 2.5 | group 2 |
| *Alseodaphne marlipoensis* (H.W. Li) H.W. Li | globose, 2.2 | group 2 |
| *Alseodaphne petiolaris* Hook. f. | oblong-ovoid, 1.3 | group 2 |
| *Alseodaphne rugosa* Merr. & Chun | oblate, 4.5 | group 2 |
| *Alseodaphne sichourensis* H.W. Li | ellipsoid, 3.0 | group 2 |
| *Alseodaphne* sp. NP | globose, 5.0 | group 2 |
| *Alseodaphne ximengensis* H.W. Li et J. Li | subglobose, 4.7 | group 2 |

References

**1.** Li, HW, PAI, PY, LEE, SK, WEI, FN, Yang, YC, Huang, PH, et al. *Lauraceae*. In: Li, HW (Ed.), Flora Reipublicae Popularis Sinicae, vol. 31. Science Press, Beijing, China; 1982.

**2.** Li, HW, Li, J, Huang, PH, Wei, FN & van der Werff, H. *Lauraceae*. In: Wu, ZY, Raven, PH, Hong, DY (Eds.), Flora of China, vol. 7. Science Press and Missouri Botanical Garden Press, Beijing, China, St. Louis, Missouri, USA; 2008.

**3**. Kostermans, AJGH. New and critical Malaysian plants IV. Reinwardtia, 1956; 4, 1-40.

**4.** Kostermans, AJGH. Lauraceae. Reinwardtia, 1957; 4, 275-277.

**5**. Kostermans, AJGH. New and critical Malaysian plants VI. Reinwardtia, 1960; 5, 341-369.

**6**. Kostermans, AJGH. Materials for a revision of Lauraceae I. Reinwardtia, 1968; 7, 291-356.

**7**. Kostermans, AJGH. Materials for a revision of Lauraceae III. Reinwardtia, 1970; 8, 21-196.

**8.** Kostermans, AJGH. A synopsis of *Alseodaphne* Nees (Lauraceae). *Candollea*, 1973; 28, 93-136.

**9**. Mo, YQ, Li, L, Zhang, JG, Li, HW & Li, J. Discovery of *Alseodaphne* *rugosa* Merr. et Chun (Lauraceae) in SE Yunnan, China and its biogeographical significance. Guihaia, 2017; 37, (In Press)

**10**. Thakur, BK, Anthwal, A, Rawat, DS, Rawat, B, Rashmi & Rawat, M. A review on genus *Alseodaphne*: phytochemistry and pharmacology. Mini-Reviews in Organic Chemistry, 2012; 9, 433-445.
