# Supplementary figures and images for: Alseodaphnopsis: A new genus of Lauraceae based on molecular and morphological evidence
Source: PLoS One. 2017 Oct 18;12(10):e0186545. doi: 10.1371/journal.pone.0186545 (PMC5646853; doi:10.1371/journal.pone.0186545)

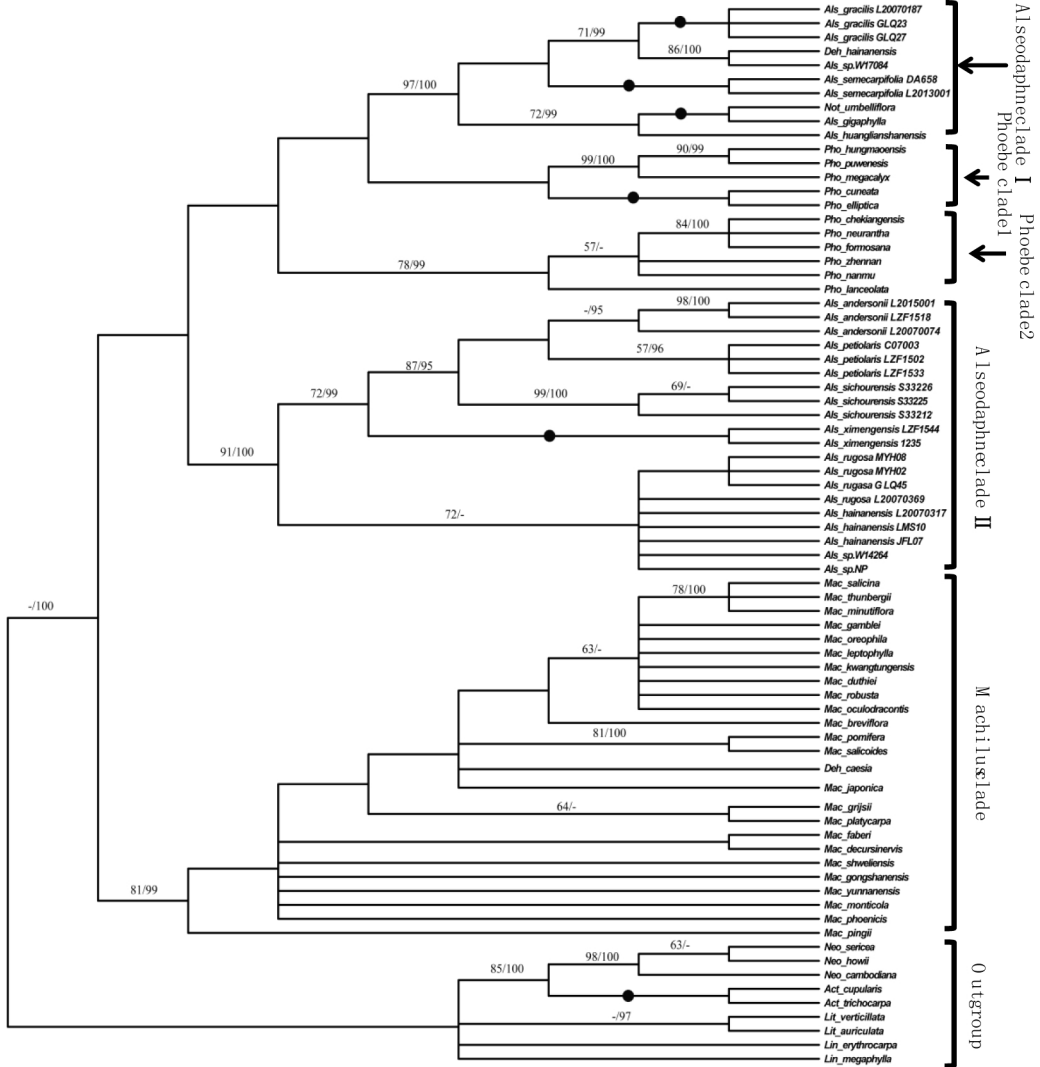

Supplement: S1 Fig — Bootstrap values (≥ 50%) / Bayesian posterior probabilities (≥ 95%) are shown above branches. ● = both bootstrap value and Bayesian posterior probability 100%. (PDF) [file pone.0186545.s004.pdf]

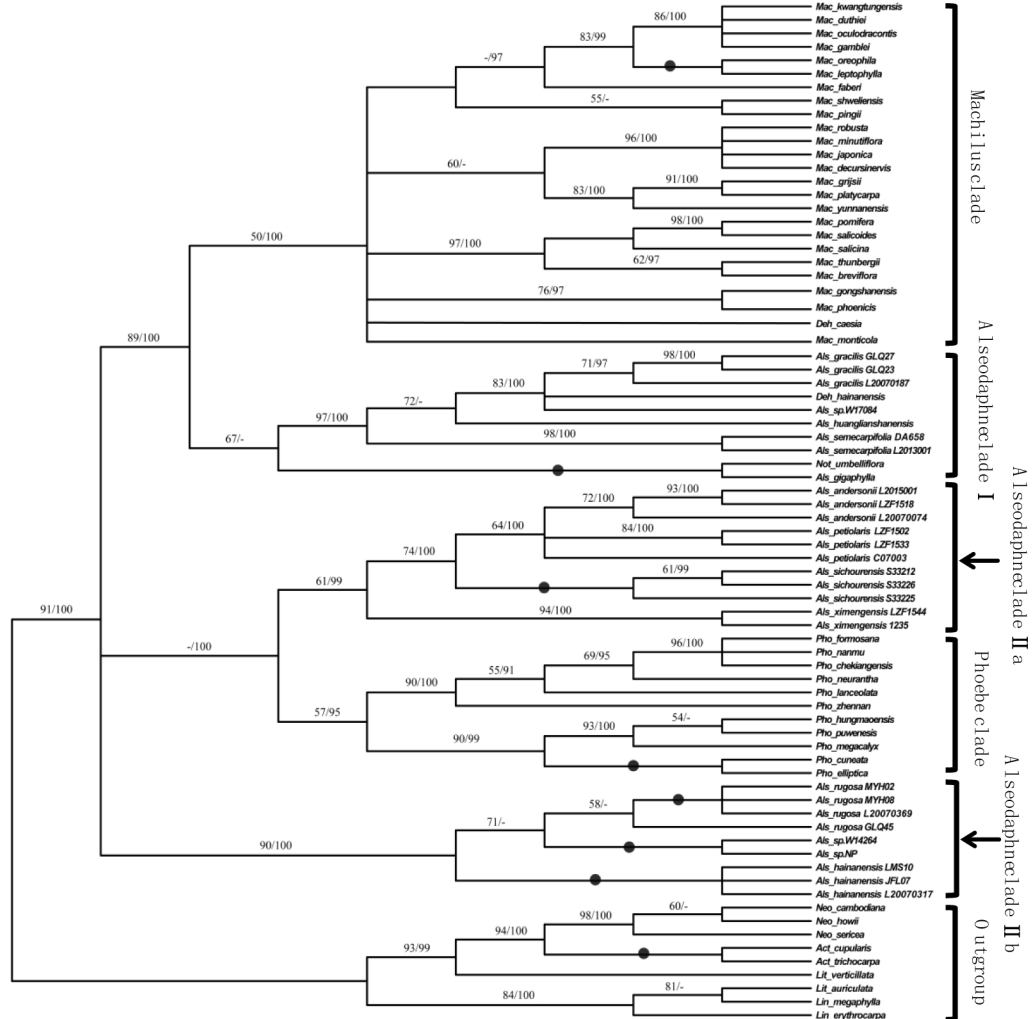

Supplement: S2 Fig — Bootstrap values (≥ 50%) / Bayesian posterior probabilities (≥ 95%) are shown above branches. ● = both bootstrap value and Bayesian posterior probability 100%. (PDF) [file pone.0186545.s005.pdf]
